# PIAS1 protects against myocardial ischemia-reperfusion injury by stimulating PPARγ SUMOylation

**DOI:** 10.1186/s12860-018-0176-x

**Published:** 2018-11-12

**Authors:** Bo Xie, Xinyu Liu, Jie Yang, Jinke Cheng, Jianmin Gu, Song Xue

**Affiliations:** 1 Department of Cardiovascular Surgery, Renji Hospital, Shanghai Jiao Tong University School of Medicine, 160 Pujian Road, Shanghai, 200127 China; 2 Department of Biochemistry and Molecular Cell Biology, Key Laboratory for Cell Differentiation and Apoptosis of Chinese Ministry of Education, Shanghai Jiao Tong University School of Medicine, 200025, 280 South Chongqing Road, Shanghai, China; 3 Department of General Surgery, Qingpu Branch of Zhongshan Hospital, Fudan University, 201700, 1158 East Gongyuan Road, Shanghai, China; 4 Core Facility of Basic Medical Sciences, Shanghai Jiao Tong University School of Medicine, 200025, 280 South Chongqing Road, Shanghai, China

**Keywords:** Ischemia-reperfusion injury, PIAS1, SUMOylation, PPARγ, NF-κB

## Abstract

**Background:**

Myocardial ischemia-reperfusion injury (IRI) has become one of the most serious complications after reperfusion therapy in patients with acute myocardial infarction. Small ubiquitin-like modification (SUMOylation) is a reversible process, including SUMO E1-, E2-, and E3-mediated SUMOylation and SUMO-specific protease-mediated deSUMOylation, with the latter having been shown to play a vital role in myocardial IRI previously. However, little is known about the function and regulation of SUMO E3 ligases in myocardial IRI.

**Results:**

In this study, we found dramatically decreased expression of PIAS1 after ischemia/reperfusion (I/R) in mouse myocardium and H9C2 cells. PIAS1 deficiency aggravated apoptosis and inflammation of cardiomyocytes via activating the NF-κB pathway after I/R. Mechanistically, we identified PIAS1 as a specific E3 ligase for PPARγ SUMOylation. Moreover, H9C2 cells treated with hypoxia/reoxygenation (H/R) displayed reduced PPARγ SUMOylation as a result of down-regulated PIAS1, and act an anti-apoptotic and anti-inflammatory function through repressing NF-κB activity. Finally, overexpression of PIAS1 in H9C2 cells could remarkably ameliorate I/R injury.

**Conclusions:**

Collectively, our findings demonstrate the crucial role of PIAS1-mediated PPARγ SUMOylation in protecting against myocardial IRI.

**Electronic supplementary material:**

The online version of this article (10.1186/s12860-018-0176-x) contains supplementary material, which is available to authorized users.

## Background

With the tremendous rise in the standard of living, acute myocardial infarction (MI) has become a common cardiovascular emergency that causes a large number of deaths in modern society. Timely and effective myocardial reperfusion appears to be the only therapeutic approach for reducing acute myocardial ischemic injury and limiting MI size [1]. However, as a direct result of blood flow restoration to the ischemic tissue, myocardial ischemia-reperfusion injury (IRI) can lead to cell death and additional cardiac dysfunction. The underlying molecular mechanisms of myocardial IRI involve inflammation, calcium overload, oxidative stress, cytokine release and infiltration of neutrophil [2]. Peroxisome proliferator-activated receptor γ (PPARγ) is a member of the nuclear receptor superfamily of ligand-inducible transcription factors, which has been shown to play a vital role in various physiological and pathological processes, including glucose and lipid metabolism, immunity and cardiovascular disease [3]. Activation of PPARγ can suppress the inflammatory response in cardiac tissue after ischemia/reperfusion (I/R) and thus alleviate ischemic pathological damage [4, 5]. In our previous study, we found that PPARγ mediates the protective effect of quercetin against myocardial IRI via suppressing the NF-κB pathway [6].

It has taken more than 20 years to identify protein modification by small ubiquitin-like modification (SUMOylation) [7]. Protein SUMOylation is a reversible process catalyzed by the activating (E1), conjugating (E2) and ligating (E3) enzymes and can be reversed by a family of SUMO-specific proteases (SENPs) [8, 9]. Only one E1 and one E2 enzyme have been reported in mammalian cells, whereas more than eight SUMO E3 ligases have been found to catalyze the transfer of SUMO from E2 UBC9 to a substrate. The protein inhibitor of activated STAT (PIAS) family of proteins [10], including PIAS1, PIAS3, PIASxα, PIASxβ and PIASy, belong to the largest group of SUMO E3 ligases characterized by an SP-RING motif [11]. The requirement of the location of a RING-finger domain in the middle of a PIAS is essential to the E3 ligase activity of PIAS proteins. Various studies have shown that PIAS-mediated SUMOylation of target proteins is involved in a wide range of cellular processes [12–16].

We have previously shown that SENP1 deficiency exacerbates IRI in cardiomyocytes via an HIF1α-dependent pathway [17], indicating the involvement of protein SUMOylation in myocardial IRI. However, it is unknown whether SUMO E3 ligases are regulated in myocardial IRI. In this study, we identify PIAS1 as a specific E3 ligase for PPARγ SUMOylation in the myocardium. PIAS1-mediated PPARγ SUMOylation protects against apoptotic and inflammatory injury by inhibiting NF-κB activation after ischemia/reperfusion. Our data suggest a potential clinical role of PIAS1 in IRI therapy.

## Results

### Expression of PIAS1 is reduced after ischemia/reperfusion in mouse myocardium and H9C2 cells

**Fig. 1:**
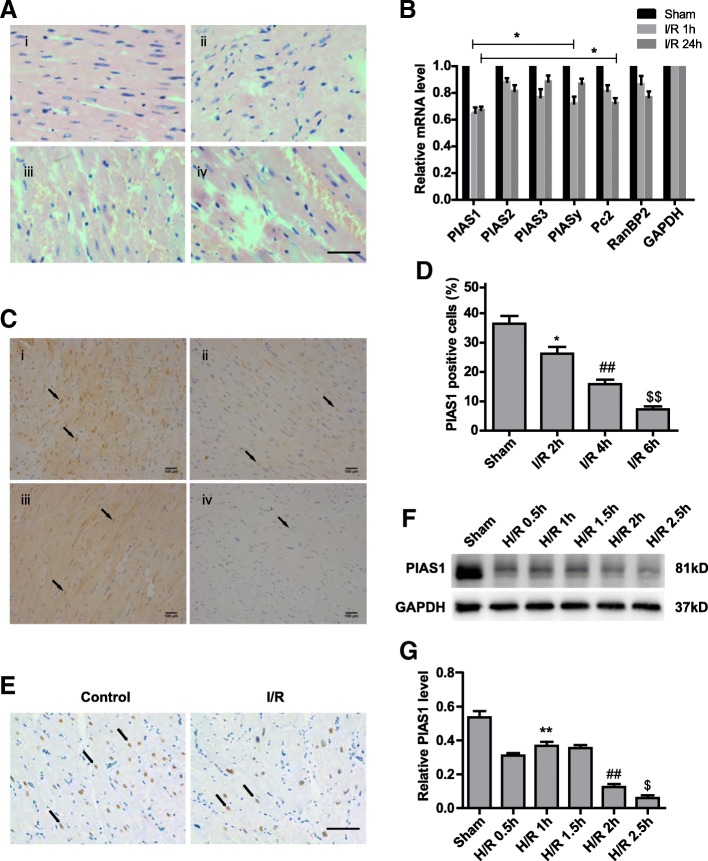
Reduced expression of PIAS1 after ischemia/reperfusion in mouse myocardium and H9C2 cells. **a** H&E staining of representative mouse myocardial sections under different treatments. (i) Sham-operated, (ii) 2 h after I/R, (iii) 4 h after I/R, and (iv) 6 h after I/R. Scale bars, 100 μm. **b** RT-PCR analysis of selected SUMO E3 ligases in mouse myocardium after I/R (*n* = 3). **c** IHC analysis of PIAS1 expression in representative mouse myocardial sections under different treatments. (i) Sham-operated, (ii) 2 h after I/R, (iii) 4 h after I/R, and (iv) 6 h after I/R. Scale bars, 100 μm. **d** Quantification of PIAS1-positive cells (arrows in **c**) in mouse myocardial sections of different groups (*n* = 5). **e** IHC analysis of PIAS1 expression in human cardiac tissues from patients under different surgeries. Scale bars, 100 μm. **f** Western blot analysis of PIAS1 proteins in H9C2 cells under normoxic condition (Sham) or hypoxia and reoxygenation (H/R) treatments. GAPDH was set as loading control. **g** Quantification of the densitometry of the western blot band in F (*n* = 3). Experiments in **a**, **b**, **e** and **f** were performed three times, and experiments in **c** and **d** were performed five times. Data presented are means ± SD, ^*^P < 0.05 vs. sham group, ^##^*P* < 0.01 vs. I/R 2 h group, and ^$$^*P* < 0.01 vs. I/R 4 h group

To address the function and regulation of SUMO E3 ligases in myocardial IRI, we developed a mouse model of cardiac ischemia and reperfusion by surgical operation as described previously [17]. Along with the extended period of reperfusion (2-6 h), we found that mouse myocardium exhibited more hypertrophy, necrosis and inflammation, and the arrangement of myofibers became more disordered compared with that of the sham-operated myocardium (Figure 1a). These results confirmed serious injury after I/R. In this mouse model. We detected the mRNA levels of known SUMO E3 ligases in the myocardium after I/R treatment. To our surprise, all of these E3 ligases were down-regulated after I/R. Here, we focused on PIAS1, which showed the largest reduction in the injured myocardium (Figure 1b, *n* = 3). We then confirmed the decreased expression of in situ PIAS1 in mouse myocardium by immunohistochemistry (IHC) after 2–6 h of reperfusion (Figure 1c and d, *n* = 5) (I/R 2 h group vs. sham group, power = 0.87; I/R 4 h group vs. I/R 2 h group, power = 0.89; I/R 6 h group vs. I/R 4 h group, power = 0.98). More importantly, PIAS1 was detected in cardiac tissues from patients undergoing cardiac surgery; as shown in Figure 1e, myocardium with ischemia and reperfusion had a much lower expression of PIAS1 than that in myocardium before clamping (control group). In addition, H9C2 cells were exposed to hypoxia for 2 h followed by an extended period of reoxygenation. We found remarkably diminished PIAS1 proteins after hypoxia/ reoxygenation (H/R) (Figure 1f and g, *n* = 3). These data reveal that PIAS1 is down-regulated in the myocardium during I/R.

### PIAS1 deficiency aggravates injury after H/R via activation of NF-κB pathway

**Fig. 2:**
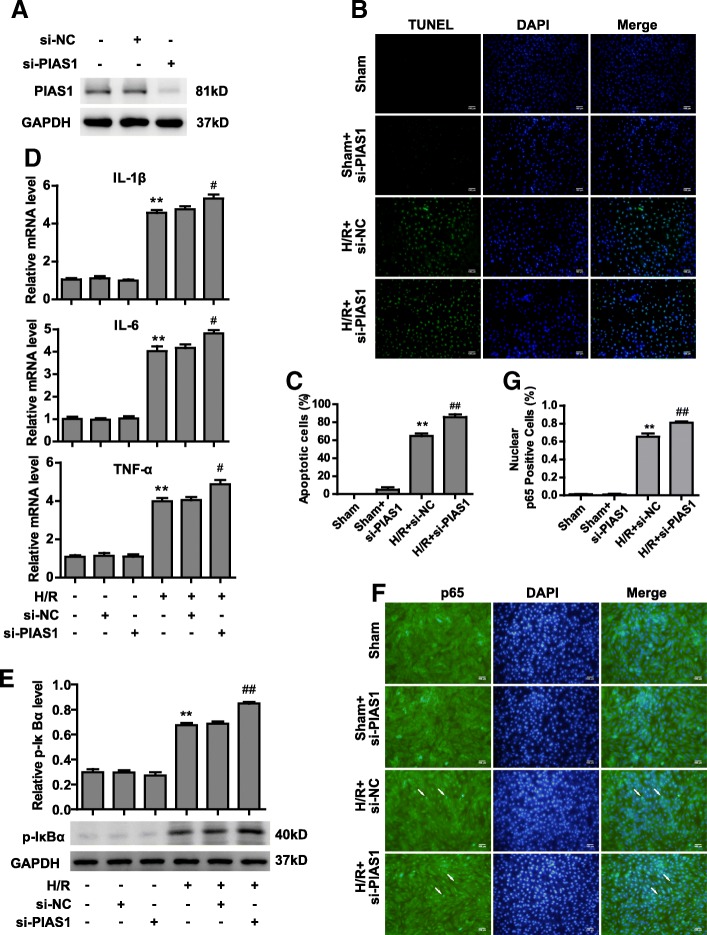
Knockdown of PIAS1 aggravates injury after H/R via activating NF-κB pathways. **a** Western blot analysis of PIAS1 proteins in H9C2 cells transfected with si-NC (non-specific siRNA) or si-PIAS1 (against PIAS1 siRNA). **b** TUNEL staining of apoptotic H9C2 cells transfected with si-NC or si-PIAS1 under normoxic condition or H/R treatments. Scale bars, 100um. **c** Quantification of apoptotic rates based on TUNEL staining (*n* = 5). **d** RT-PCR analysis of IL-1β, IL-6 and TNFα in H9C2 cells transfected with si-NC or si-PIAS1 under normoxic condition or H/R treatment (*n* = 3). **e** Western blot analysis of phosphorylated IκBα proteins in H9C2 cells transfected with si-NC or si-PIAS1 under normoxic condition or H/R treatments. Quantification of the densitometry of the western blot band is shown above (*n* = 3). **f** Representative immunofluorescence images of p65 proteins (Green) in H9C2 cells transfected with si-NC or si-PIAS1 under normoxic condition or H/R treatments. DAPI indicates cell nucleus. Scale bars, 100um. **g** Quantification of nuclear p65-positive H9C2 cells based on TUNEL staining (*n* = 5). Experiments in **a**, **d** and **e** were performed three times, and experiments in **b**, **c**, **f** and **g** were performed five times. Data presented are means ± SD, ^#^*P* < 0.05 vs. H/R + si-NC group, ^**^P < 0.01 vs. sham group, and ^##^*P* < 0.01 vs. H/R + si-NC group

PIAS1 has five highly conserved functional domains, with different functions: the SAP (scaffold attachment factor-A/B, Acinus and PIAS), the PINIT motif, the RING-type zinc-binding domain, the SBD (SUMO binding domain, also indicated as SIM, SUMO interacting motif) and a C-terminal serine/threonine rich region. The RING domain is essential for the E3 SUMO-ligase activity of PIAS1, especially for the catalytic C351 and W372 sites, which is highly conserved in human, mouse and rat species (Additional file 1: Figure S1). To investigate the physiological function of PIAS1 in IRI, endogenous PIAS1 was knocked down in H9C2 cells by transfecting with siRNA (Figure 2a). Silencing of PIAS1 did not affect cell death under normoxia. Hypoxia and reoxygenation increased the apoptotic rate of negative control (si-NC) cells to approximately 60%, whereas PIAS1 deficiency increased the apoptotic rate to more than 80% (Figure 2b and c, *n* = 5). Inflammation is an important contributor to the pathophysiology of cardiac IRI [18]. Thus, we tested the mRNA levels of inflammatory cytokines in H9C2 cells. As shown in Figure 2d (*n* = 3), knockdown of PIAS1 significantly promoted the expression of IL-1β, IL-6 and TNFα compared with that of si-NC cells after H/R. It is well known that activating the NF-κB pathway is critical to facilitating myocardial inflammation during I/R [19]. H9C2 cells exposed to H/R displayed more phosphorylated IκBα (p-IκBα) and nuclear translocation of the NF-κB p65 subunit, two major indicators of NF-κB activation [20]. Lack of PIAS1 led to increased levels of p-IκBα and nuclear p65 compared with those observed in si-NC cells after H/R (Figure 2e, *n* = 3, f and g, *n* = 5). Collectively, on the account of the reduced expression of PIAS1 in cardiomyocytes after I/R, PIAS1 deficiency can partially aggravate apoptosis and inflammation in a manner by activating NF-κB pathways.

### PIAS1 enhances PPARγ SUMOylation by its SUMO E3 ligase activity

**Fig. 3:**
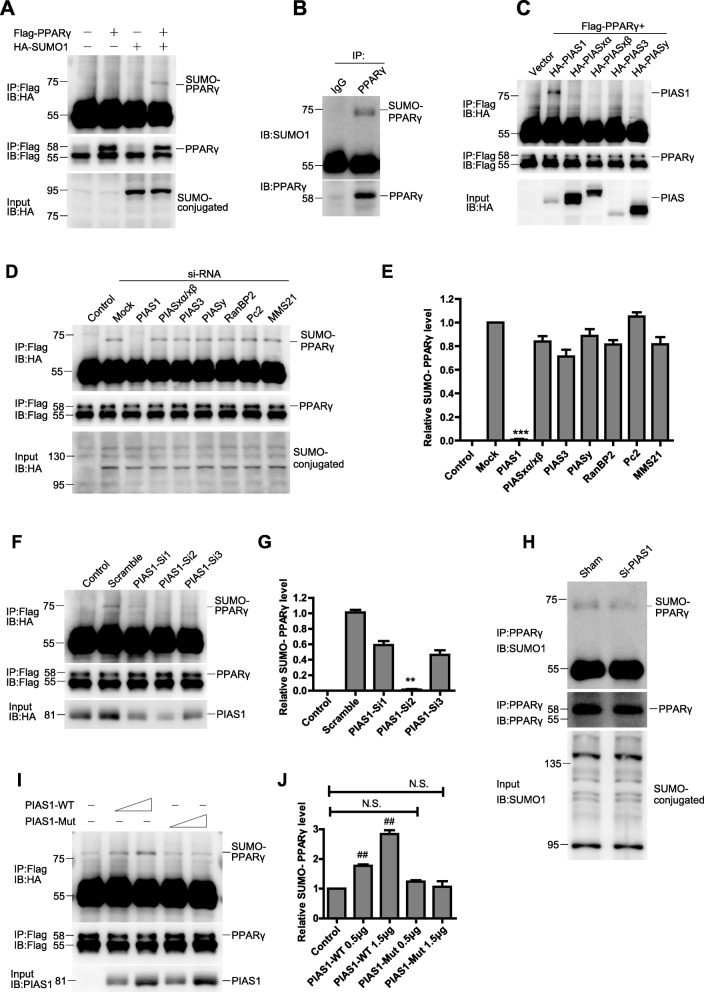
PIAS1 promotes PPARγ SUMOylation by its SUMO E3 ligase activity. **a** 293 T cells were cotransfected with Flag-PPARγ and HA-SUMO1 plasmids as indicated. The cell lysates were immunoprecipitated (IP) with anti-Flag antibody, followed by blotting (IB) with anti-HA or anti-Flag antibody. Whole-cell lysates were blotted (IB) with anti-HA antibody. **b** H9C2 cell lysates were immunoprecipitated with anti-PPARγ antibody, followed by blotting with anti-SUMO1 or anti-PPARγ antibody. **c** 293 T cells were cotransfected with vector, Flag-PPARγ and HA-tagged SUMO E3 ligases plasmids as indicated. The cell lysates were immunoprecipitated with anti-Flag antibody, followed by blotting with anti-HA or anti-Flag antibody. Whole-cell lysates were blotted with anti-HA antibody. **d** 293 T cells were cotransfected with Flag-PPARγ, HA-SUMO1 and siRNAs against selected SUMO E3 ligases as indicated (control cells were transfected without HA-SUMO1). The cell lysates were immunoprecipitated with anti-Flag antibody, followed by blotting with anti-HA or anti-Flag antibody. Whole-cell lysates were blotted with anti-HA antibody. **e** Quantification of the densitometry of the SUMO-PPARγ band in D (*n* = 3). **f** 293 T cells were cotransfected with Flag-PPARγ, HA-SUMO1 and siRNAs against PIAS1 as indicated (control cells were transfected without HA-SUMO1). The cell lysates were immunoprecipitated with anti-Flag antibody, followed by blotting with anti-HA or anti-Flag antibody. Whole-cell lysates were blotted with anti-PIAS1 antibody. **g** Quantification of the densitometry of the SUMO-PPARγ band in **f** (*n* = 3). **h** H9C2 cells were transfected with siRNAs against PIAS1. Cell lysates were immunoprecipitated with anti-PPARγ antibody, followed by blotting with anti-SUMO1 or anti-PPARγ antibody. Whole-cell lysates were blotted with anti-SUMO1 antibody. **i** 293 T cells were cotransfected with Flag-PPARγ, HA-SUMO1 and increasing doses of PIAS1 wild-type (PIAS1-WT) or PIAS1 catalytic mutant (PIAS1-Mut) as indicated. The cell lysates were immunoprecipitated with anti-Flag antibody, followed by blotting with anti-HA or anti-Flag antibody. Whole-cell lysates were blotted with anti-PIAS1 antibody. **j** Quantification of the densitometry of the SUMO-PPARγ band in I (*n* = 3). All these experiments were performed three times. Data presented are means ± SD, ^***^P < 0.001 vs. mock group, ^**^P < 0.01 vs. scramble group, and ^##^*P* < 0.01 vs. control group

As our previous study demonstrated that PPARγ protects against myocardium IRI via suppressing the NF-κB pathway [6], we next asked whether PPARγ was the SUMOylated target of PIAS1 in myocardium IRI. First, we confirmed PPARγ SUMOylation in 293 T cells transfected with Flag-PPARγ and HA-SUMO1. The band of SUMO1 conjugated PPARγ was present at ~ 72 kDa (Figure 3a). Then, we successfully detected endogenous PPARγ SUMOylation in H9C2 cells by immunoprecipitating (IP) with anti-PPARγ antibody (Figure 3b). To identify the specific interaction between PPARγ and known SUMO E3 ligases, we co-transfected Flag-PPARγ and HA-tagged SUMO E3 ligases into 293 T cells; as shown in Figure 3c, PPARγ only bound to PIAS1. Moreover, we used an siRNA approach against known SUMO E3 ligases, SUMO1 conjugation on PPARγ was dramatically diminished in the lane of si-PIAS1 compared with that in other lanes (Figure 3d and e, *n* = 3). The ability of PIAS1 to down-regulate PPARγ SUMOylation was further demonstrated by three additional PIAS1 siRNAs (Figure 3f and g, *n* = 3). Consistently, the PIAS1 silencing almost completely abrogated the endogenous PPARγ SUMOylation in H9C2 cells (Figure 3h). To further determine whether this effect requires PIAS1 E3 ligase activity, we mutated the E3 catalytic domain of PIAS1 and compared its activity toward SUMOylate PPARγ with that of wild-type (WT) PIAS1. As shown in Figure 3i and j (*n* = 3), overexpression of PIAS1-WT, but not that of PIAS1 catalytic-inactivation mutant (PIAS1-Mut), in 293 T cells could enhance PPARγ SUMOylation, indicating the essential role of E3 ligase activity.

### PPARγ SUMOylation antagonizes injury after H/R by suppressing NF-κB activation

**Fig. 4:**
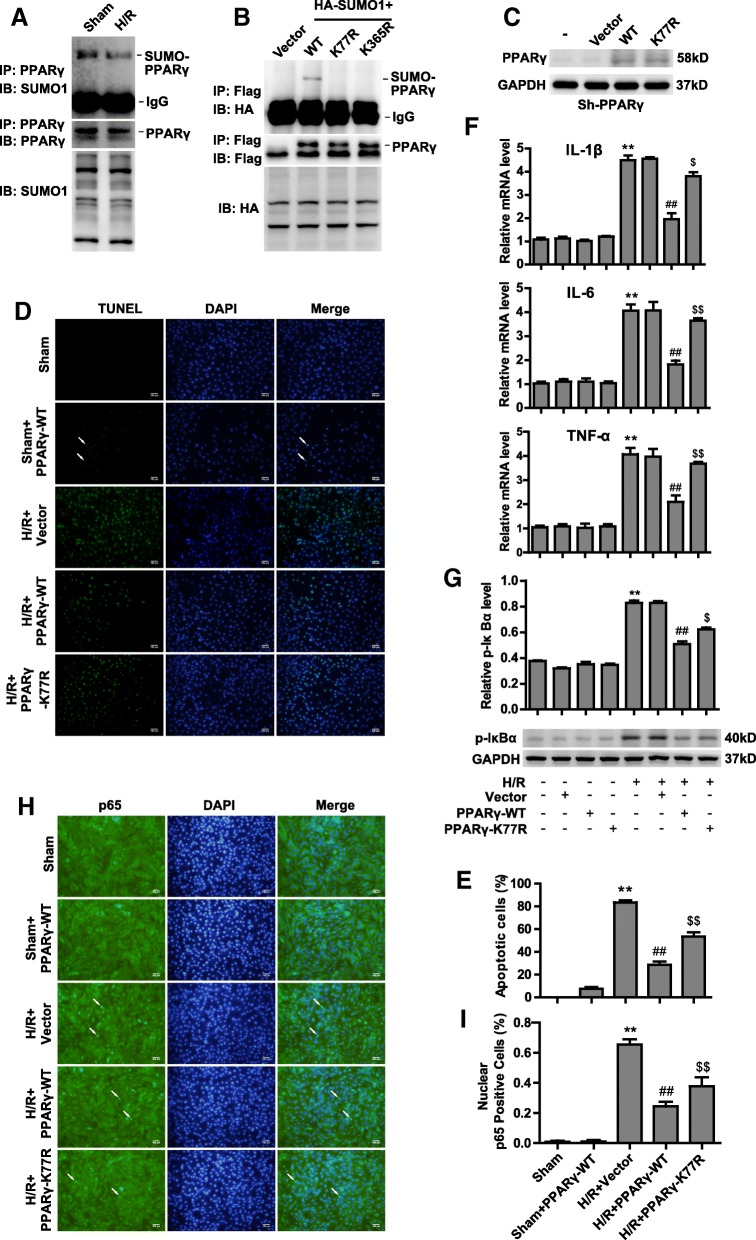
PPARy SUMOylation antagonizes injury after H/R by suppressing NF-κB activation. **a** H9C2 cells were cultured under normoxic or H/R conditions, and cell lysates were then immunoprecipitated with anti-PPARγ antibody, followed by blotting with anti-SUMO1 or anti-PPARγ antibody. Whole-cell lysates were blotted with anti-SUMO1 antibody. **b** 293 T cells were cotransfected with HA-SUMO1 and Flag-PPARγ wild-type or SUMO site mutants (K77R and K365R) plasmids as indicated. The cell lysates were immunoprecipitated with anti-Flag antibody, followed by blotting with anti-HA or anti-Flag antibody. Whole-cell lysates were blotted with anti-HA antibody. **c** PPARγ-knockdown H9C2 cells were constructed by sh-RNA lentivirus. Sh-PPARγ cells were transfected with vector, PPARγ-WT or K77R plasmids as indicated; then, cells were cultured under normoxic or H/R conditions. Cells lysates were blotted with anti-PPARγ or anti-GAPDH antibody. **d** TUNEL staining of apoptotic H9C2 cells transfected with vector, PPARγ-WT and K77R plasmids as indicated under normoxic or H/R conditions. Scale bars, 100um. **e** Quantification of apoptotic rates based on TUNEL staining (*n* = 5). **f** RT-PCR analysis of IL-1β, IL-6 and TNFα in sh-PPARγ cells transfected with vector, PPARγ-WT and K77R plasmids as indicated after being subjected to normoxic or H/R conditions (*n* = 3). **g** Western blot analysis of phosphorylated IκBα protein in sh-PPARγ cells transfected with vector, PPARγ-WT and K77R plasmids as indicated after being subjected to normoxic or H/R conditions. Quantification of the densitometry of the western blot band is shown above (*n* = 3). **h** Representative immunofluorescence images of p65 proteins (green) in sh-PPARγ cells transfected with vector, PPARγ-WT and K77R plasmids as indicated under normoxic or H/R conditions. Scale bars, 100um. **i** Quantification of nuclear p65-positive cells based on TUNEL staining (*n* = 5). Experiments in **a**, **b**, **c**, **f** and **g** were performed three times, and experiments in **d**, **e**, **h** and **i** were performed five times. Data presented are means ± SD, ^**^P < 0.01 vs. sham group, ^##^*P* < 0.01 vs. H/R + Vector group, and ^$$^*P* < 0.01 vs. H/R + PPARy-WT group

To investigate whether the protective function of PIAS1 in IRI relies on PPARγ SUMOylation, we tested the physiological role of PPARγ SUMOylation in myocardium under H/R. H9C2 cells were incubated under normoxia or H/R conditions, followed by co-IP assays with anti-PPARγ antibody. Our data showed decreased PPARγ SUMOylation after H/R treatment (Figure 4a). We confirmed lysine 77 and 365 as the SUMO sites of PPARγ [3] by transfecting wild-type or mutated PPARγ into 293 T cells. As shown in Figure 4b, SUMO1-conjugated PPARγ was remarkably abrogated when lysine 77 and 365 were mutated into arginine (K77R and K365R). We further transfected PPARγ-WT or PPARγ-K77R plasmids into PPARγ-knockdown (sh-PPARγ) H9C2 cells to exclude the endogenous PPARγ (Figure 4c). Intriguingly, PPARγ-WT could reduce the apoptotic rate to a much greater extent than could PPARγ-K77R under H/R treatment (Figure 4d and e, *n* = 5). Consistent with our previous findings, the expression levels of IL-1β, IL-6 and TNFα were significantly decreased in PPARγ-WT cells compared with those in PPARγ-K77R cells after H/R (Figure 4f, *n* = 3). We also found that PPARγ-WT, but not K77R, dramatically inhibited the phosphorylation of IκBα induced by H/R (Figure 4g, *n* = 3). In agreement, the number of nuclear p65-positive cells was much lower among PPARγ-WT cells than among PPARγ-K77R cells after H/R (Figure 4h and i, *n* = 5). Taken together, PPARγ SUMOylation along with PIAS1 plays a profitable role in resisting IRI.

### Ectopic expression of PAIS1 alleviates IRI via PPARγ SUMOylation

**Fig. 5:**
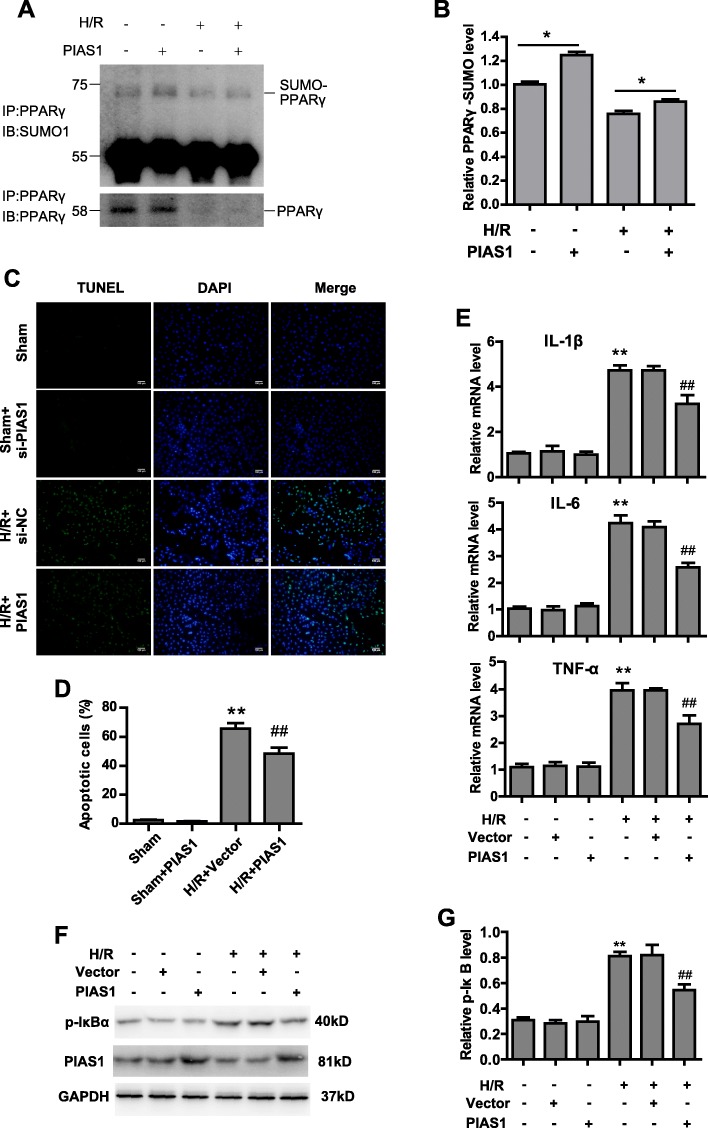
Ectopic expression of PIAS1 alleviates IRI via PPARy SUMOylation. **a** H9C2 cells were transfected with PIAS1 plasmids as indicated; then, cells were cultured under normoxic or H/R conditions. Cell lysates were immunoprecipitated with anti-PPARγ antibody, followed by blotting with anti-SUMO1 or anti-PPARγ antibody. **b** Quantification of the densitometry of the SUMO-PPARγ band in A (*n* = 3). **c** TUNEL staining of apoptotic H9C2 cells transfected with vector or PIAS1 under normoxic condition or H/R treatments. Scale bars, 100um. **d** Quantification of apoptotic rates based on TUNEL staining (*n* = 5). **e** RT-PCR analysis of IL-1β, IL-6 and TNFα in H9C2 cells transfected with vector or PIAS1 under normoxic condition or H/R treatment (*n* = 3). **f** H9C2 cells were transfected with PIAS1 plasmids as indicated; then, cells were cultured under normoxic or H/R conditions. Cells lysates were blotted with anti-PIAS1, anti- p-IκBα or anti-GAPDH antibody. **g** Quantification of the densitometry of the p-IκBα band in F (*n* = 3). Experiments in **a**, **b**, **e**, **f** and **g** were performed three times, and experiments in **c** and **d** were performed five times. Data presented are means ± SD, ^##^*P* < 0.01 vs. H/R + vector group, and ^**^P < 0.01 vs. sham group

In light of our finding that PIAS1 is essential for PPARγ SUMOylation, which protects against IRI via inhibiting the NF-κB pathway, we speculate that ectopic expression of PIAS1 in myocardium is beneficial, as the endogenous PIAS1 is down-regulated after H/R. Indeed, overexpression of PIAS1 in H9C2 cells could remarkably accumulate PPARγ SUMOylation under both normoxia and H/R conditions (Figure 5a and b, *n* = 3). Meanwhile, enforced PIAS1 significantly attenuated apoptosis of cardiomyocytes after H/R (Figure 5c and d, *n* = 5). The expression of inflammatory cytokine were also decreased after H/R by ectopic expression of PIAS1 (Figure 5e, *n* = 3). Moreover, phosphorylated IκBα was decreased in cells overexpressing PIAS1 after H/R, indicating lower activation of NF-κB (Figure 5f and g, *n* = 3). These data reveal the potential clinical function of PIAS1 in IRI therapy.

## Discussion

Protein SUMOylation has been considered as a vital regulator of cellular function in physiology and pathology. Recently, Ubc9, the E2 ligase for SUMOylation, has been found to play an essential role in isoflurane preconditioning-induced tolerance against cerebral ischemia-reperfusion injury [21]. Moreover, SENP1 protects neurons by inhibiting apoptosis during transient brain ischemia/reperfusion [22], consistently with our previous finding in myocardial IRI [17]. In this study, we analyzed the expression of SUMO E3 ligases during myocardial IRI using a mouse model. Among these E3 ligases, PIAS1 demonstrated the largest extent of reduction in myocardium after I/R. Notably, PIAS1 proteins undergo fast decline during the initial period of reperfusion, whereas the mRNA level of PIAS1 at 24 h of reperfusion becomes nearly equal to that at 1 h of reperfusion. These results imply that there may be a mechanism involved in up-regulating PIAS1 expression after long-term reperfusion. It is important and valuable to further explore this mechanism under the pathological process of myocardial IRI, as ectopic expression of PIAS1 can alleviate the injury. Recent studies have suggested that PIAS1 serves as an anti-inflammatory factor in adipose tissue and the lung [23, 24]. In agreement, our findings verify that PIAS1 antagonizes inflammation in myocardium through inhibition of the NF-κB pathway.

Our previous finding demonstrated that SENP1 protects against IRI in cardiomyocytes via a HIF1α-dependent pathway [17]. On the other hand, here we highlight the important role of PIAS1-mediated PPARγ SUMOylation in protecting against myocardial IRI, indicating us that two opposite functional proteins may have the same contribution to a cellular process through different pathways. PPARγ has been shown to benefit cardiovascular disease therapies, such as those pertaining to ventricular hypertrophy, cardiac remodeling and acute myocardial infarction [25–27]. SUMOylation of PPARγ at lysine 77 in the transactivation domain blocks its transcriptional activity, possibly by promoting co-repressor recruitment [28–30], while PPARγ is also SUMOylated at lysine 365, which in macrophages results in its occupation of the promoters of inflammatory genes and inhibition of their expression [31]. Our previous work demonstrated that PPARγ protects against myocardial IRI by suppressing the NF-κB pathway. In this study, we find that PIAS1-mediated SUMOylation of PPARγ is essential for the inactivation of NF-κB signaling. To evaluate the function of PPARγ SUMOylation in myocardial IRI, PPARγ-WT or K77R mutant was re-expressed into H9C2 cells lacking endogenous PPARγ. Unlike PPARγ-WT, K77R mutant failed to inhibit NF-κB activation effectively and alleviate inflammation and apoptosis sharply after H/R. However, we found that K77R mutant can partially rescue the phenotype of PPARγ deficiency, suggesting that the existing SUMOylation of PPARγ at lysine 365 may also play a role in regulating NF-κB activity. Further investigation is required to reveal the function of lysine 365 SUMOylation in myocardial IRI.

## Conclusions

**Fig. 6:**
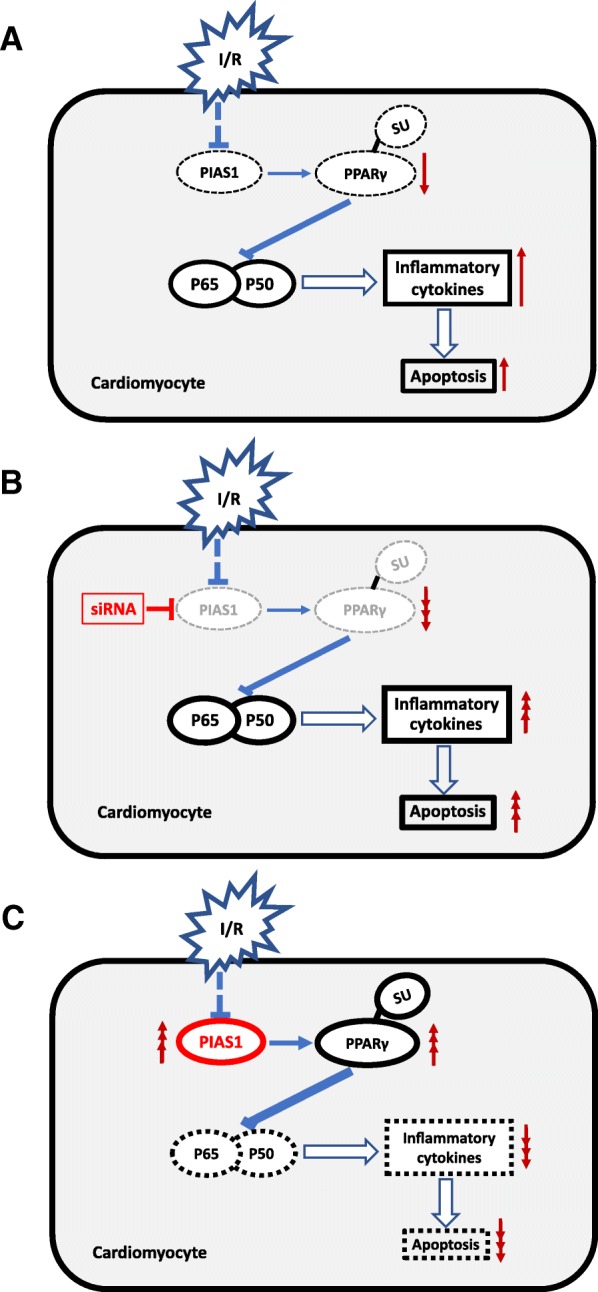
A simplified model depicting the mechanisms of PIAS1 involved in myocardial IRI. **a** PIAS1 expression is down-regulated in cardiomyocytes during ischemia/reperfusion (I/R), resulting in decreased SUMOylation of PPARγ, which directly inhibits NF-κB pathway. The activation of NF-κB can induce transcription of inflammatory cytokines and further lead to apoptosis and cell death. **b** Knockdown of PIAS1 by si-RNA can aggregate the inflammation and apoptosis of cardiomyocyte through PPARγ SUMOylation mediated NF-κB pathway during I/R. **c** Overexpression of PIAS1 alleviates injury of myocardial I/R by increasing SUMOylation of PPARγ and down-regulation of NF-κB pathway

In conclusion, our findings demonstrate the crucial role of PIAS1-mediated PPARγ SUMOylation in protecting against myocardial IRI. Up-regulation of PIAS1 may become a new therapeutic strategy to protect myocardium from IRI (Figure 6).

## Methods

### IRI in human myocardium

Twelve male patients with an average age of 64.83 ± 3.326 years were diagnosed with moderate mitral stenosis without other physical illnesses, and underwent mitral valve replacement under cardiopulmonary bypass. The patients are comparable. Mitral valve replacement surgery which requires the use of extracorporeal circulation can simulate the process of myocardial ischemia and reperfusion, refer to our previous publication [17]. We acquired myocardial tissue at two time points. The first time was before the aorta was clamped (Sham) and the second time was 15 min after unclamping(IRI). The specimens were immediately washed in cold phosphate-buffered saline (PBS) and then preserved in 4% paraformaldehyde. All patients signed informed consent. This research conformed to the Declaration of Helsinki and was approved by the Institutional Review Board of Renji Hospital, Shanghai Jiao Tong University School of Medicine.

### Animals and IRI in mouse myocardium

Male C57/BL6 mice (8 weeks old; weight, 22-24 g) were purchased at the Xipuer-Bika Experimental Animal Center (Shanghai, China). Mice were housed in the Animal Experimental Center of Shanghai Jiao Tong University School of Medicine. Mice were randomly divided into four groups: [1] sham-operation group (Sham, *n* = 6); [2] ischemia 30 min and reperfusion 2 h group (I/R 2 h, *n* = 6); [3] ischemia 30 min and reperfusion 4 h group (I/R 4 h, *n* = 6); [4] and ischemia 30 min and reperfusion 6 h group (I/R 6 h, *n* = 6). Mouse heart IRI surgery was performed as described [6]. All animal research were performed in accordance with NIH guidelines (Guide for the Care and Use of Laboratory Animals) and the Use Committee of Shanghai Jiao Tong University.

### RNA isolation and quantitative RT-PCR analysis

Total RNA was isolated from cells or tissues by using Trizol reagent (Invitrogen). cDNAs were reverse transcribed with 1 μg of RNA using the Prime Script™ RT reagent kit (Takara RR037A). Fluorescence real-time quantitative PCR was performed on the LC480 system (Roche) with SYBER Green Supermix (Takara RR420A) according to the manufacturer’s procedures. Values were normalized to GAPDH. The relative gene expression level was calculated using the comparative Ct method formula: 2^-ΔΔCt^. Primers used in this study are listed in Table 1.

**Table 1:** Sense and reverse primers

Genes	Species	F/R	Primers
PIAS1	Mouse	F	5’-GCGGACAGTGCGGAACTAAA-3’
		R	5’-ATGCAGGGCTTTTGTAAGAAGT-3’
PIAS2	Mouse	F	5’-TTCCCTTATTCCAGTTGATCCCC-3’
		R	5’-CCACTGCTGGTTATGACCCC-3’
PIAS3	Mouse	F	5’-CAGCTCAGATTCTGTCTCTGTG-3’
		R	5’-TTCTTGGTTGGAGGGAGGTAA-3’
PIASy	Mouse	F	5’-GGAGGCCAAAAACATGGTGAT-3’
		R	5’-GGGCTACAGTCGAACTGCAC-3’
RanBP2	Mouse	F	5’-GCTGGCTGCATTGTGCTATC-3’
		R	5’-GTGGGCCATCGTTTCCAGG-3’
Pc2	Mouse	F	5’-AAGAAGCGGATACGCAAGGG-3’
		R	5’-GGAGGAGTCTTGAAGCCCAG-3’
GAPDH	Mouse	F	5’-AGGTCGGTGTGAACGGATTTG-3’
		R	5’-TGTAGACCATGTAGTTGAGGTCA-3’
IL-1β	Rat	F	5’-CTGTGTCTTTCCCGTGGACC-3’
		R	5’-CAGCTCATATGGGTCCGACA-3’
IL-6	Rat	F	5’-GAACAACGATGATGCACTTGCAG-3’
		R	5’-CCTTAGCCACTCCTTCTGTGAC-3’
TNF-α	Rat	F	5’-CCAGTGTGGGAAGCTGTCTT-3’
		R	5’-AAGCAAAAGAGGAGGCAACA-3’
GAPDH	Rat	F	5’-TGTGTCCGTCGTGGATCTGA-3’
		R	5’-TTGCTGTTGAAGTCGCAGGAG-3’

### Immunohistochemical and immunofluorescence analysis

In brief, Human and mouse heart tissues were harvested and fixed with 4% paraformaldehyde, then were dehydrated, paraffin-embedded, and sectioned (5 μm) prior to staining. Heart sections were exposed to 3% hydrogen peroxide for 10 min and treated for 20 min in boiling 0.01 M citric acid (pH 6.0), then blocked with bovine serum albumin for 1 h. Sections were subsequently incubated with anti-PIAS1 antibody (1:200 diluted in PBS) overnight at 4 °C, and anti-rabbit IgG secondary antibody (1:1000 diluted in PBS) were incubated for 2 h at room temperature. The avidin–biotin complex (ABC) and 3,3′-diaminobenzidine tetrahydrochloride (DAB) were then incubated, nuclei were stained with hematoxylin. Images were acquired using Nikon microscope. For PIAS1 positive cell measurement, the number of positive cells per mm^2^ tissue section were counted in 10 random visual fields under high magnification. Immunofluorescence analysis of NF-κB was performed as described previously [6].

### Immunoprecipitation (IP) and western blot analysis

Cells were lysed in IP buffer (50 mM Tris-HCl, pH 7.4, 400 mM NaCl, 0.5% sodium deoxycholate, 0.3% Triton X-100, 0.1% SDS, 10 mM N-ethylmaleimide, and protease inhibitors) for30 min on the ice, then cell lysates were sonicated and centrifuged at 20000×g for 10 min at 4 °C. The supernatants were transferred to new tubes. The appropriate antibodies and protein A/G beads were added for 6 h at 4 °C, beads were then washed with IP buffer and eluted in 1% SDS solution. Proteins were detected by western blot analysis, antibodies used in this study were PIAS1 (ab32119), inducible nitric oxide synthase (iNOS) (ab15323), GAPDH (ab70699) and IgG (ab210935) from Abcam, Anti-PPARγ (sc-7273) from Santa Cruz Biotechnology, Anti-Flag (F3165) from Sigma, Anti-HA (MMS-101P) from Covance. Anti-SUMO1 (18–2306) from Zymed, Cyclooxygenase-2 (COX-2) (12282), NF-κBp65 (8242), and phospho-IκBα (p-IκBα) (2859) from Cell Signaling Technology.

### Cell culture

HEK-293 T cells and myocardial H9C2 cells were cultured in Dulbecco’s modified Eagle’s medium (4.5 g/L D-glucose) supplemented with 10% FBS. For normal condition (sham), all cells were maintained at 37 °C in a humidified 5% CO_2_ incubator. For hypoxia and reoxygenation (H/R), cells were cultured in an airtight incubation tank for 4 h with a < 1% oxygen concentration followed by hours of reoxygenation as indicated. The cultured H9C2 cells were randomly divided into different groups. siRNAs as indicated were transfected into H9C2 cells for 48 h before treatments, plasmids as indicated were transfected into H9C2 for 24 h before treatments.

### Plasmids and RNAi

pcDNA3-Flag-PIAS1, HA-PIAS1, HA-PIASxα, HA-PIASxβ, HA-PIAS3, HA-PIASy, HA-SUMO-1, and Flag-PPARγ plasmids were kindly provided by Jinke Cheng (Shanghai Jiao Tong University School of Medicine, Shanghai, China). Flag-PPARγ-K77R and Flag-PPARγ-K365R were generated using PCR-based mutagenesis. siRNAs against PIAS1, PIASxα/xβ, PIAS3, PIASy, RanBp2, Pc2, MMS21 and nonspecific control siRNA were designed and synthesized by GenePharma (Shanghai, China). The siRNA sequences are listed in Table 2.

**Table 2:** Sequences of siRNA

Genes	Species	Sense/Anti	siRNA sequences
PIAS1-Si1	Human	Sense	5’-GGCCCUUACAUGUUCUCAUTT-3’
		Antisense	5’-AUGAGAACAUGUAAGGGCCTT −3’
PIAS1-Si2	Human	Sense	5’-GUGCGGAACUAAAGCAAAUTT −4’
		Antisense	5’-AUUUGCUUUAGUUCCGCACTT −4’
PIAS1-Si3	Human	Sense	5’-CUCCAUAUGAACACCUUAUTT 3’
		Antisense	5’-AUAAGGUGUUCAUAUGGAGTT −3’
Negative control (NC)	Human	Sense	5’-GGACACCUGCUCGUUACUUTT-3’
		Antisense	5’-AAGUAACGAGCAGGUGUCCTT-3’
PIAS2	Human	Sense	5’-GGAAUCCACUCGUUGCCUUTT-3’
		Antisense	5’-AAGGCAACGAGUGGAUUCCTT-3’
PIAS3	Human	Sense	5’-CCAGGUGCUUCUUGGCUUUTT-3’
		Antisense	5’-AAAGCCAAGAAGCACCUGGTT-3’
PIASy	Human	Sense	5’-GGAGUAAGAGUGGACUGAATT-3’
		Antisense	5’-UUCAGUCCACUCUUACUCCTT-3’
RanBP2	Human	Sense	5’-GCGCGAAAUUGUUUCGUUUTT-3’
		Antisense	5’-AAACGAAACAAUUUCGCGCTT-3’
Pc2	Human	Sense	5’-GCACUAACCGAGAGAAAUATT-3’
		Antisense	5’-UAUUUCUCUCGGUUAGUGCTT-3’
MMS21	Human	Sense	5’-GGACAAGGCAAUGGUUGAATT-3’
		Antisense	5’-UUCAACCAUUGCCUUGUCCTT-3’
PIAS1	Rat	Sense	5’-GCAAAUGGUUAUGAGCCUUTT-3’
		Antisense	5’-AAGGCUCAUAACCAUUUGCTT-3’
Negative control (NC)	Rat	Sense	5’-UUCUCCGAACGUGUCACGUTT-3’
		Antisense	5’-ACGUGACACGUUCGGAGAATT-3’

### Terminal deoxynucleotidyl transferase-mediated dUTP end labeling (TUNEL) assay

The TUNEL assay was performed using the in situ Cell Death Detection kit (Beyotime Biotechnology, Shanghai, China) according to the manufacturer’s instructions. Images were acquired using Zeiss 710 microscope; The percentage of TUNEL-positive cells/total cells was calculated in 10 visual fields chosen randomly from each section for statistical analysis.

### Statistical analysis

All experiments were performed at least three times. All data were presented as mean ± standard deviation. Graphs and statistical comparisons were performed using GraphPad Prism Software. Parametrical data were compared using Student’s t-test or one-way ANOVA analysis to analyze significance (*P < 0.05; **P < 0.01).

## Additional file

Additional file 1: **Figure S1.** RING domain of PIAS1 in human, rat and mouse. The PIAS1 protein composes of 651 amino acids and has five distinct functional domains: the SAP (scaffold attachment factor-A/B), the PINIT motif, the RING-type zinc-binding domain, the SBD (SUMO binding domains) and a C-terminal serine/threonine rich region. The SAP domain is involved in direct DNA or protein binding, The RING domain is essential for the E3 SUMO-ligase activity of PIAS1, especially for the catalytic C351 and W372 sites, which is highly conserved in human, mouse and rat species. (JPG 22 kb)
