# Supplementary figures and images for: PIAS1 protects against myocardial ischemia-reperfusion injury by stimulating PPARγ SUMOylation
Source: BMC Cell Biol. 2018 Nov 12;19:24. doi: 10.1186/s12860-018-0176-x (PMC6233564; doi:10.1186/s12860-018-0176-x)

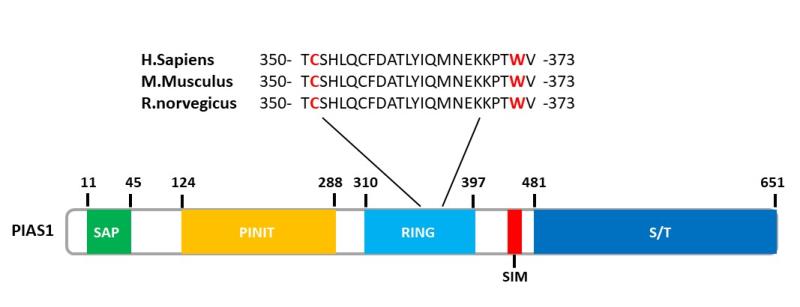

Supplement: Supplementary file 1 — Figure S1. RING domain of PIAS1 in human, rat and mouse. The PIAS1 protein composes of 651 amino acids and has five distinct functional domains: the SAP (scaffold attachment factor-A/B), the PINIT motif, the RING-type zinc-binding domain, the SBD (SUMO binding domains) and a C-terminal serine/threonine rich region. The SAP domain is involved in direct DNA or protein binding, The RING domain is essential for the E3 SUMO-ligase activity of PIAS1, especially for the catalytic C351 and W372 sites, which is highly conserved in human, mouse and rat species. (JPG 22 kb) [file 12860_2018_176_MOESM1_ESM.jpg]
